# Do anthropogenic disturbances affect the stress physiology and immunity of *Liolaemus cuyumhue*? The case of an endangered lizard from the Monte Desert of Patagonia

**DOI:** 10.1242/bio.062777

**Published:** 2026-10-06

**Authors:** María V. Brizio, Fernando Duran, Facundo Cabezas-Cartes, Luciano J. Avila, Jorgelina M. Boretto

**Affiliations:** ^1^ Grupo de Herpetología Patagónica, Instituto Patagónico para el Estudio de Ecosistemas Continentales (IPEEC-CONICET), Abuelas de Plaza de Mayo 2915, 9120 Puerto Madryn, Chubut, Argentina; ^2^ Laboratorio de Ecofisiología e Historia de vida de Reptiles, Instituto de Investigaciones en Biodiversidad y Medio Ambiente (INIBIOMA-CONICET), Universidad Nacional del Comahue, Quintral 1250, San Carlos de Bariloche, 8400 Río Negro, Argentina; ^3^ Instituto de Investigación en Paleobiología y Geología (IIPG, CONICET-UNRN), Av. Roca 1242, Gral. Roca, 8332 Río Negro, Argentina

**Keywords:** Corticosterone, Leukocytes, Liolaemidae, Oil and gas exploitation, Stress

## Abstract

In arid environments, hydrocarbon exploitation causes habitat alterations, affecting the availability of microhabitats for thermoregulation, predator evasion, and foraging opportunities. This is the first study to evaluate the effects of this anthropogenic disturbance on stress and immunological parameters in a liolaemid lizard, *Liolaemus cuyumhue*, an endemic and critically endangered species (Monte Desert, Patagonia, Argentina). During the spring–summer seasons, we compared the leukocyte profiles, heterophil-to-lymphocyte (H/L) ratio, capture body temperatures (*T*_b_), serum corticosterone concentrations (CORTs), and body condition index (BCI) of adults from a site disturbed by hydrocarbon exploitation and an undisturbed site. We found that lymphocyte percentages were higher in lizards from the disturbed site, whereas heterophils, basophils, and monocytes were more abundant at the undisturbed site. The H/L differed significantly between sites and seasons, being higher at the undisturbed site and during spring. A significant negative association between the H/L ratio and *T*_b_ was observed only at the disturbed site. CORT measured in adult males varied seasonally, being higher in summer, but showed no differences between sites. Our results suggest that individuals that survived and persisted in the disturbed site have been able to cope with environmental conditions, which at present do not appear to induce chronic physiological stress.

## Teaser

**Summary:** This is the first study that evaluates the effects of anthropogenic environmental disturbance on the stress physiology and immunological parameters of an endemic and critically endangered lizard from the Monte Desert (Patagonia, Argentina).

## INTRODUCTION

Habitat loss and degradation are among the main causes of decline and extinction of wildlife species worldwide (Fahrig, 2003; Tan et al., 2023). Species with limited vagility, high ecological specialization, or marked physiological sensitivity are often particularly vulnerable to these changes, as they exhibit less flexibility to adapt to modified or fragmented environmental conditions (French et al., 2010; Simpson et al., 2023). In some areas, such as North Patagonia of Argentina, hydrocarbon exploitation is one of the main sources of environmental disturbance in arid and semiarid ecosystems inhabited by numerous vertebrate species. Activities associated with oil exploration and extraction, such as road construction, pipeline installation, soil movement, heavy machinery use, and waste generation, directly alter habitat structure (Mazzonia and Vázquez, 2009; Pérez et al., 2023) and habitat quality and consequently produce profound alterations in the ecological and energetic dynamics of wild populations (Haddad et al., 2015).

Habitat modifications that include soil compaction and loss of vegetation cover may affect the availability and quality of trophic resources, alter predator abundance and distribution, and disrupt social or reproductive interactions among animals (Tuomainen and Candolin, 2011; Tilman et al., 2014). Particularly in reptiles, those habitat modifications also change the availability of shelters and thermal microclimates for thermoregulation (Keinath et al., 2017; Brizio et al., 2025). These environmental alterations act as both direct and indirect stressors, increasing the energetic costs associated with thermoregulation, foraging, and predator avoidance (Wingfield, 2013; Winfree et al., 2015). Consequently, animals chronically exposed to degraded or fragmented environments may experience a reduction in body condition, which serves as an integrative indicator of the energetic balance between resource acquisition and expenditure (Peig and Green, 2009, 2010). A decline in body condition not only reflects a compromised nutritional state but can also be associated with suboptimal physiological performance, reduced reproductive success, and a lower ability to cope with stressors and immunological challenges (Romero and Wikelski, 2002; Labocha and Hayes, 2012; Fernández et al., 2025).

The physiological response to environmental stressors is mediated by the activation of the hypothalamic–pituitary–adrenal axis, leading to the release of glucocorticoids, such as corticosterone (e.g. Romero, 2004; Davis and Maney, 2018; Duran et al., 2024). These hormones regulate energy balance and mobilization but can also influence immune function when elevated for prolonged periods (Sapolsky et al., 2000). The increase in glucocorticoid levels generally results in changes in circulating leukocyte profiles, characterized by an increase in heterophils and a decrease in lymphocytes (Davis et al., 2008; Silvestre, 2014; Gangloff et al., 2017). This glucocorticoid-mediated redistribution of leukocytes reflects a shift in the allocation of immune resources, enhancing innate immune components while suppressing adaptive responses. This mechanism is thought to enhance short-term survival during acute stress but may compromise immune competence if sustained over time (Sapolsky et al., 2000; Dhabhar, 2014). Consequently, the heterophil-to-lymphocyte (H/L) ratio is widely used as an indirect and integrative indicator of long-term stress responses in reptiles and other vertebrates, rather than of the rapid fluctuations in blood glucocorticoid levels (Cockrem, 2013; Davis and Maney, 2018; Lamar et al., 2024). The incorporation of hematological assessments alongside endocrine measurements provides useful tools for evaluating stress physiology and overall health in reptiles inhabiting environments with varying degrees of anthropogenic disturbance (Sykes and Klaphake, 2008; Goessling et al., 2016; Chamut and Arce, 2018).

Studies of squamate lizards have provided evidence for an association between leukocyte profiles and glucocorticoid concentrations, highlighting their value as indicators of immune function and physiological stress. Experimental and laboratory studies have demonstrated that acute stressors, such as confinement (e.g. lizard *Plestiodon inexpectatus*, Seddon and Klukowski, 2012), repeated handling for ecophysiological experiments, or repeated blood sampling (e.g. *Liolaemus attenboroughi*, Duran et al., 2024), can increase both the H/L ratio and corticosterone concentrations. Also, experimental manipulation of corticosterone levels has allowed assessment of its impact on overall physiological condition and immune function (e.g. *Lacerta vivipara*, Meylan et al., 2010). However, fewer studies have examined how anthropogenic disturbance affects these immune and endocrine responses under natural conditions (e.g. *Urosaurus ornatus*, French et al., 2008; Galápagos marine iguana *Amblyrhynchus cristatus*, French et al., 2010; *Thamnophis sirtalis*, Gangloff et al., 2017). Available evidence suggests that environmental disturbance may shape stress physiology and immune function in complex, context-dependent ways (French et al., 2008, 2010; Gangloff et al., 2017).

Given that reptiles rely heavily on microhabitat characteristics to regulate body temperature, avoid predation, forage and engage in social and reproductive interactions, anthropogenic disturbances may compromise their behavioral performance, physiological balance, and, ultimately, population persistence (Brizio et al., 2025). Therefore, the combined assessment of morphometric, endocrine, and hematological parameters constitutes a comprehensive physiological approach to understanding how anthropogenic pressures could affect the health and viability of reptile populations. The model species of the present study, the Añelo Sand Dunes lizard (*Liolaemus cuyumhue*, Avila et al., 2009), is a critically endangered lizard (Avila, 2016), endemic to the dune environments of the Monte Desert in Neuquén Province, Argentina, where it faces severe threats. Extensive oil and gas exploitation has fragmented the habitat and altered vegetation structure and microhabitat thermal conditions (Brizio et al., 2021, 2025), potentially compromising the persistence of *L. cuyumhue* populations.

The objective of this study was to determine the effects of environmental disturbance caused by hydrocarbon exploitation on the immune and stress physiology throughout the activity season of the lizard *L. cuyumhue*. We hypothesized that (1) anthropogenic environmental disturbance alters body condition, endocrine physiology, and immune responses and (2) these traits display seasonal variation throughout the activity period of *L. cuyumhue*. We predict that body condition, serum corticosterone concentration (CORT), and leukocyte profiles of *L. cuyumhue* will differ between sites, with low body conditions, high CORTs, and high H/L ratios in lizards from the disturbed site, reflecting chronic stress-induced immunosuppression and energetic demands of physiological stress responses. Also, we predict that the CORT and H/L ratio will be the highest during reproductive or energetically demanding periods in both sites, whereas body condition will be the lowest during this period, recovering before the hibernation period.

## RESULTS

### Effects of site on leukocyte profile

The most frequent cells in the leukocyte profile were lymphocytes, followed by heterophils, in both the disturbed and undisturbed sites (Table 1, Fig. 1). The percentages of other cell types, such as basophils and monocytes, were comparatively low, whereas eosinophils and azurophils were the least abundant in both sites (Table 1, Fig. 1). Significant differences in leukocyte types were detected between the sites: lymphocyte percentages were higher at the disturbed site, whereas heterophils, basophils, and monocytes were more abundant at the undisturbed site (Table 1). Eosinophils and azurophils showed no significant differences between sites (Table 1).

**Fig. 1.**
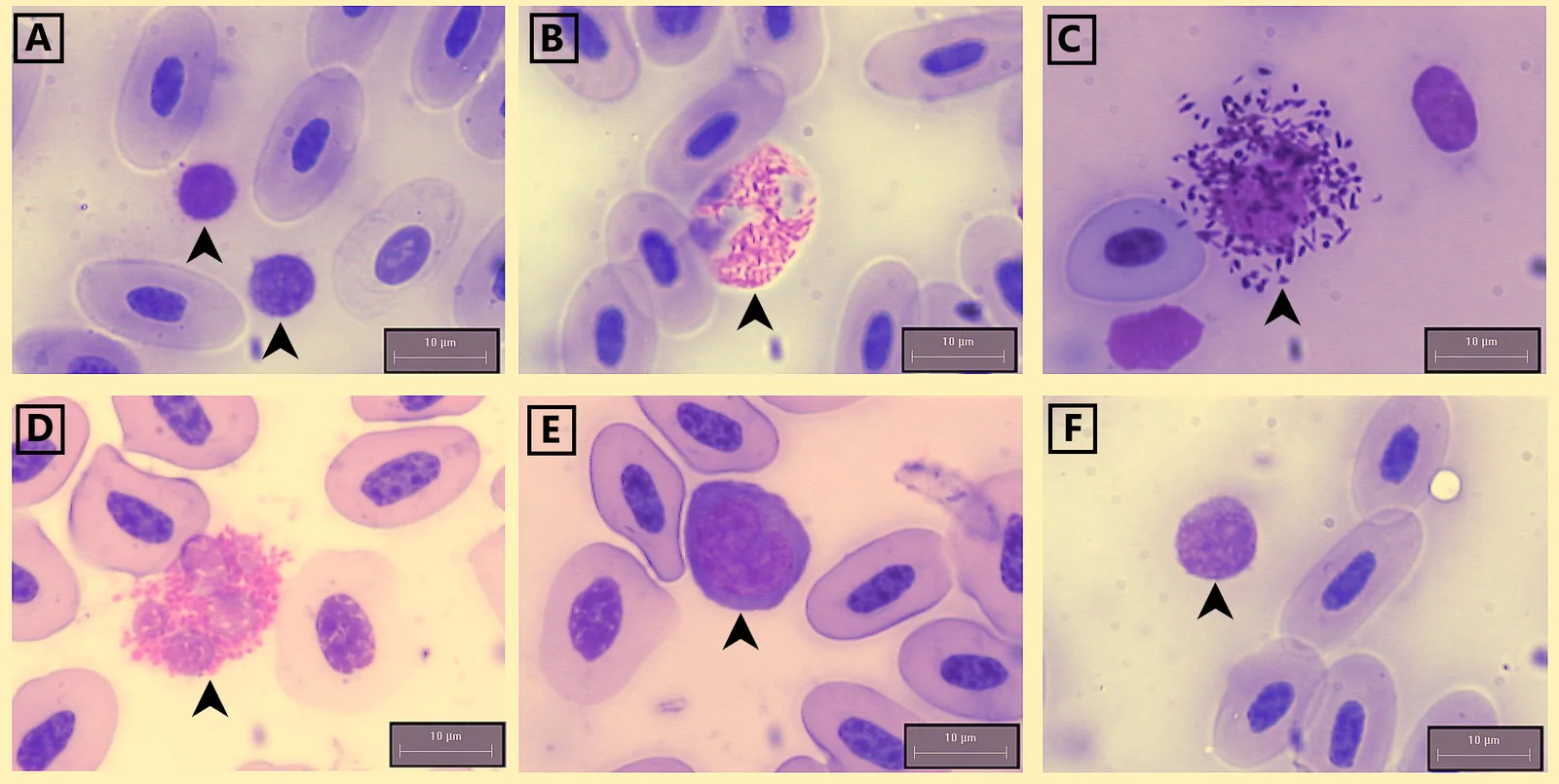
**Light micrographs of leukocyte types in *L. cuyumhue*.** Lymphocyte (A), heterophil (B), basophil (C), eosinophil (D), monocyte (E), and azurophil (F) are shown. May-Grünwald Giemsa stain. Scale bars: 10 μm.

**Table 1.** Percentages of each leukocyte type (heterophils, eosinophils, basophils, lymphocytes, monocytes, and azurophils) and the H/L ratio are presented for the leukocyte profile (based on a count of 100 white blood cells)

	Undisturbed site *N*=53		Disturbed site *N=*46			
Leukocyte profile	Median	Range	Median	Range	Mann–Whitney *U* test	*P*
Lymphocytes (%)	64.00	28–92	72.00	35–87	*U*=497.500	<0.001*
Heterophils (%)	20.00	3–54	16.50	4–35	*U*=706.500	0.001*
Basophils (%)	9.00	2–31	6.50	2–33	*U*=875.500	0.016*
Eosinophils (%)	0.00	0–1	0.00	0–15	*U*=1199.000	0.793
Monocytes (%)	8.00	0–19	3.00	0–13	*U*=409.500	<0.001*
Azurophils (%)	0.00	0–1	0.00	0–2	*U*=1212.500	0.933
H/L	0.333	0.033–1.742	0.227	0.057–0.642	*U*=658.500	<0.001*

Comparisons between undisturbed and disturbed sites in *L. cuyumhue* are shown. Sample sizes (*N*), median, ranges, and results of the Mann–Whitney *U* test are also provided. Significant *P-*values are indicated with an asterisk (*).

Analysis of the effects of site, season, and sex on the H/L ratio revealed a significant effect of both site [generalized linear model (GLM): likelihood ratio test (LRT): χ^2^_1,96_=10.92, *P*=0.001] and season (LRT: χ^2^_1,96_=5.10, *P*=0.024) on H/L (Fig. 2A), whereas no significant effect of sex was observed (LRT: χ^2^_1,96_=0.33, *P*=0.567). In terms of effect size, the H/L ratio was significantly lower at the disturbed site (H/L_mean_=0.250±0.017) than at the undisturbed site (H/L_mean_=0.340±0.022; Fig. 2A). The estimated mean H/L ratio in the undisturbed site was 1.36 times higher than that in the disturbed site (ratio=1.36±0.13, *P*=0.0013; Table 2). A significant seasonal effect was also observed, with a lower H/L ratio in summer (H/L_mean_=0.261±0.016) than in spring (H/L_mean_=0.325±0.024; Fig. 2A), independently of site and sex. Mean H/L ratios were 1.24 times higher in spring than in summer (ratio=1.24±0.12, *P*=0.026; Table 2).

**Fig. 2.**
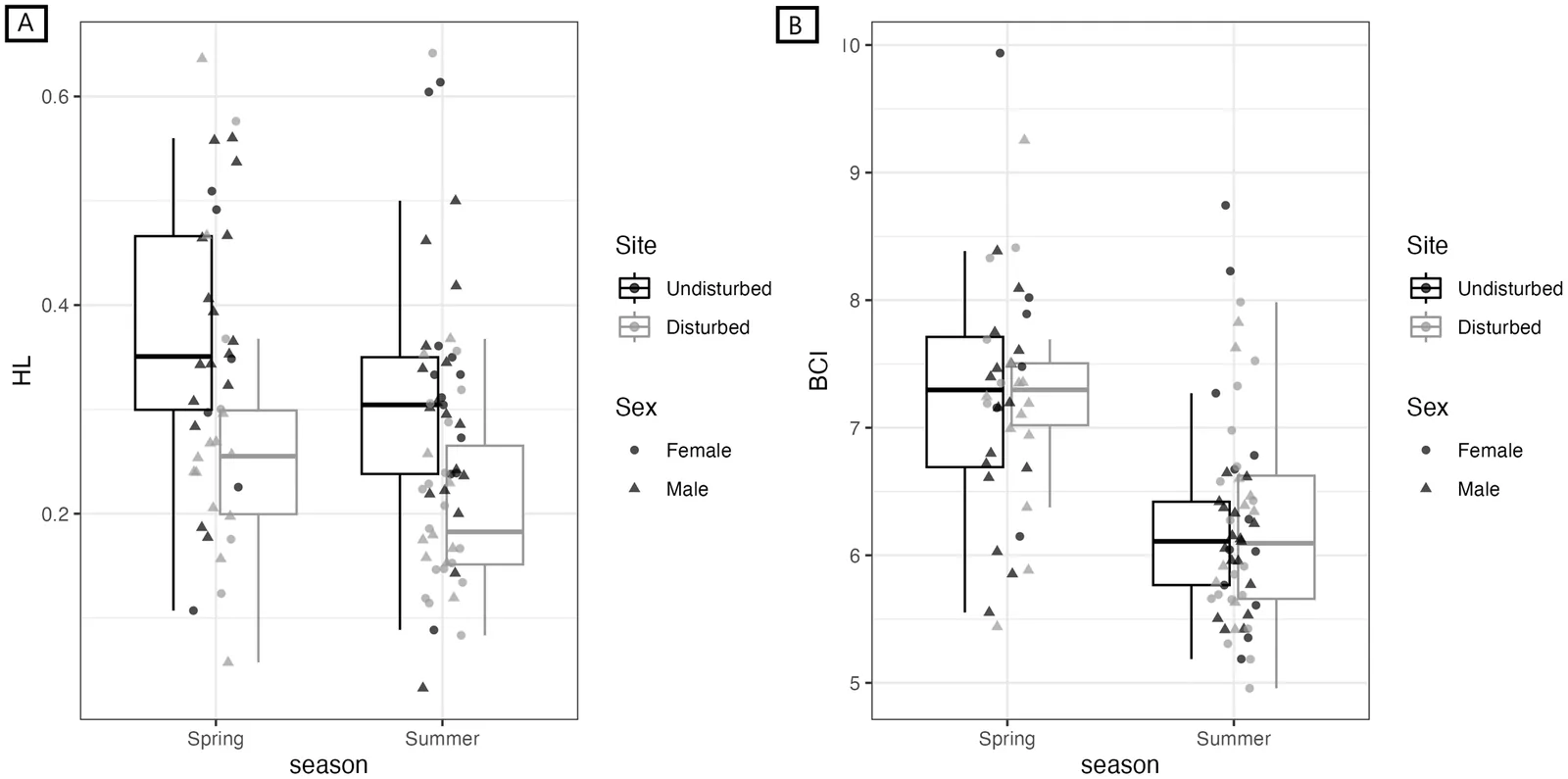
**Seasonal variation in (A) H/L ratio and (B) BCI of *L. cuyumhue* from undisturbed and disturbed sites in the Monte Desert, Argentina.** Boxplots show the median and interquartile range, and points represent individual observations, differentiated by sex (females • and males ▴).

**Table 2.** Descriptive data of study variables considering season, sex, and site in *L. cuyumhue*

	Group	H/L	*N*	*T*_b_	*N*	BCI	*N*	CORT males	*N*
Season	Spring – undisturbed	**0.359**	24	34.89±0.46	24	7.292±0.196	24	3.99±0.71	11
	Spring – disturbed	**0.256**	18	35.65±0.74	18	7.284±0.206	18	**7.35**	14
	Summer – undisturbed	0.309±0.02	29	35.54±0.56	20	**6.102**	29	4.40±1.34	7
	Summer – disturbed	**0.183**	28	37.15±0.53	27	6.254±0.157	28	6.39±0.54	8
Sex	Males – undisturbed	**0.341**	34	35.03±0.43	31	6.544±0.136	34		
	Males – disturbed	**0.239**	23	36.28±0.66	23	6.788±0.190	23		
	Females – undisturbed	**0.333**	19	35.49±0.67	15	7.007±0.298	19		
	Females – disturbed	**0.208**	23	36.83±0.60	22	6.526±0.218	23		

Means (±s.e.) or medians (in bold), as appropriate, for each variable, and sample sizes (*N*) are presented.

On the other hand, the H/L ratio was significantly negatively correlated with body temperature (*T*_b_) at the disturbed site (Pearson correlation, *r*_disturbed_*=*−0.349, *P*=0.019, *N*=45), but not at the undisturbed site (simple linear regression, *F*_1,42 undisturbed_*=*0.857, *P*=0.360, *N*=44; descriptive data presented in Table 2).

### Effects of site on CORT in males

Mean CORT was 5.29 ng/ml at the undisturbed site (range=0.20–8.24 ng/ml; *N*=25) and 5.46 ng/ml at the disturbed site (range=0.20–8.59 ng/ml; *N*=15). CORT was not associated with snout–vent length (SVL; Pearson correlation, *r*_disturbed_=0.187, *P*=0.504; *r*_undisturbed_=0.060, *P*=0.774), body mass (simple linear regression, *F*_1,14 disturbed_=0.513, *P*=0.486; Pearson correlation, *r*_undisturbed_=0.014, *P*=0.948), or *T*_b_ (Pearson correlation, *r*_disturbed_=−0.037, *P*=0.896; simple linear regression, *F*_1,20 *un*disturbed_=2.674, *P*=0.118) in adult males at either site (Table 2). In addition, none of the leukocyte types (heterophils, lymphocytes, basophils, eosinophils, monocytes, or azurophils) showed a significant correlation with CORT in males at either the undisturbed or disturbed site (Spearman correlations, all *P>*0.05).

The analysis of the effects of season and the interaction between site and H/L ratio on CORT revealed a significant interaction between site and H/L ratio (GLM: LRT χ^2^_1,39_=5.83, *P*=0.016), indicating that the relationship between H/L and CORT differed across sites (Fig. 3). In the undisturbed site, the H/L ratio was not significantly associated with CORT (β=−0.33±0.98, *P*=0.737; Fig. 3). In contrast, in the disturbed site, the relationship was strongly negative, as indicated by the significant interaction term (β=−5.53±1.80, *P*=0.004), meaning that individuals with higher H/L ratios exhibited lower CORTs at this site (Fig. 3). Season also had a significant effect on CORT (LRT χ^2^_1,39_=4.38, *P*=0.036), with higher CORT in summer than in spring (β=0.35±0.17, *P*=0.048; descriptive data presented in Table 2).

**Fig. 3.**
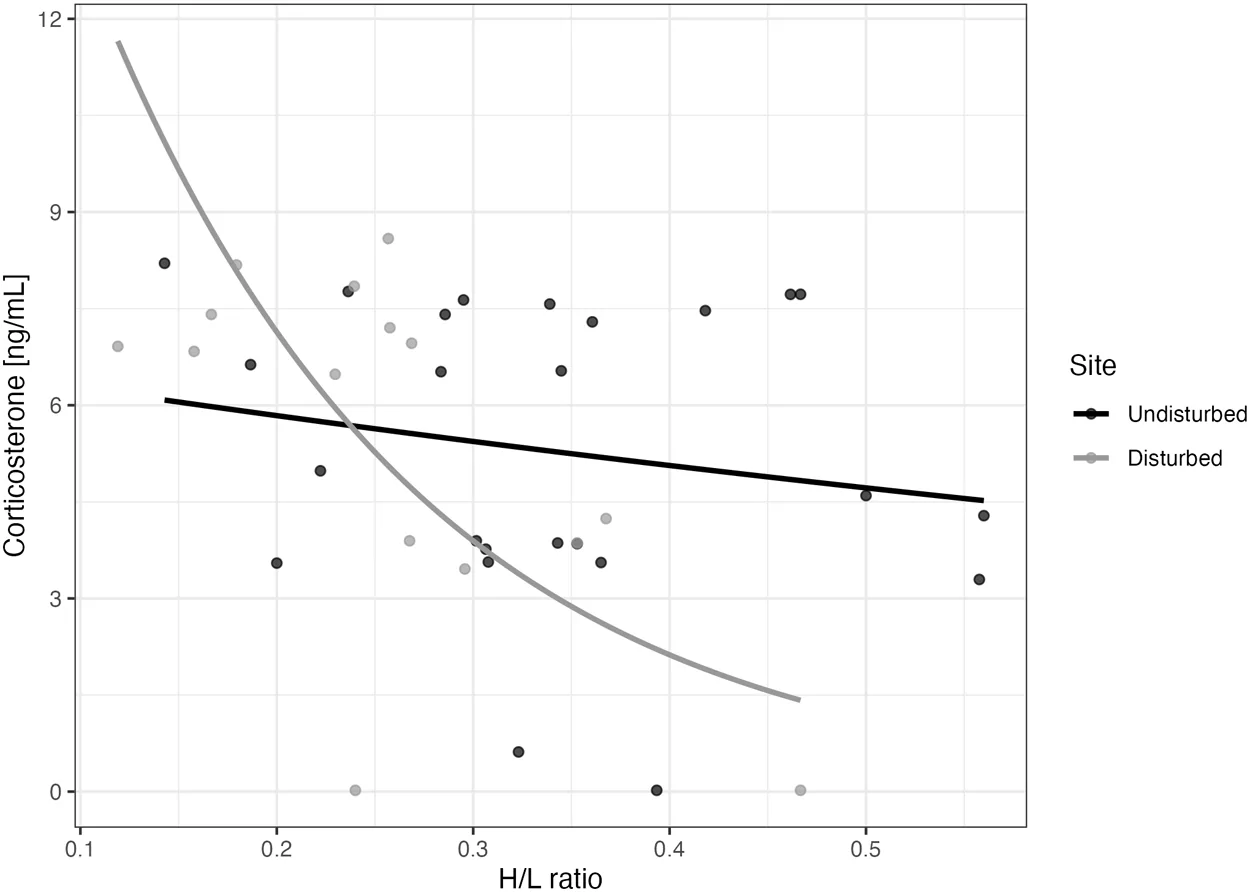
**Relationship between the H/L ratio and CORT in *L. cuyumhue* from disturbed and undisturbed sites.** Points indicate observed data, and solid lines represent fitted values from the GLM (Gamma distribution, log link function).

### Effects of site on body condition index (BCI)

The analysis of the effects of season (spring–summer) and site on BCI showed a significant effect of season (GLM: LRT: χ^2^_1,96_=33.19, *P*<0.001), whereas site had no significant effect on BCI (LRT: χ^2^_1,96_=0.03, *P*=0.859; Fig. 2B). BCI was higher in spring than in summer, with mean values of 7.26±0.15 and 6.24±0.11, respectively (Fig. 2B). This corresponds to a ratio of means of 1.16 (s.e.=0.031, *P*<0.001), indicating that individuals had, on average, 16% higher body condition in spring (descriptive data presented in Table 2).

A significant positive correlation was found between BCI and H/L ratio at the undisturbed site (Pearson correlation, *r=*0.282, *P*=0.041, *N*=53), whereas no significant correlation was detected at the disturbed site (*r=*0.225, *P*=0.113, *N*=46). Finally, CORT in males showed no significant relationship with BCI at the undisturbed site (simple linear regression, *F*_1,23 undisturbed_=0.19, *P*=0.670, *N*=25) and no significant correlation at the disturbed site (Pearson correlation, *r*_disturbed_=−0.001, *P*=0.996, *N*=15; Table 2).

## DISCUSSION

The present study addresses the effects of environmental disturbance caused by anthropogenic activities of gas and oil exploitation on the immune and stress physiology of a critically endangered lizard species, *L. cuyumhue*. By analyzing leukocyte profiles, CORTs, body temperature, and body condition in adult lizards from disturbed and undisturbed sites throughout the activity season, we evaluated how both extrinsic factors, such as seasonality and environmental disturbance, and intrinsic factors, including sex, stress status, and nutritional state, may influence selected immunological and endocrine parameters. *L. cuyumhue* from the disturbed site presented a higher lymphocyte percentage in blood, whereas heterophils, basophils, and monocytes were more abundant in lizards from the undisturbed site. The effect of environmental context was significant, and a higher H/L ratio was found in lizards from the undisturbed site.

Several studies have shown that when animals experience stress, the relative proportion of heterophils increases in circulation, while the proportion of lymphocytes declines, leading to an overall rise in the H/L ratio (reviewed in Davis and Maney, 2018). This pattern is conserved across vertebrates and therefore can be a reliable way to index stress levels (reviewed in Davis et al., 2008; Davis and Maney, 2018). The higher H/L ratio observed in *L. cuyumhue* lizards from the undisturbed site compared to those from the disturbed site contradicts our hypothesis. However, individuals that have managed to survive and persist in the degraded environment may represent a subset that has successfully coped with or undergone acclimatization to the new conditions. An alternative hypothesis is that the low H/L ratio may reflect a diminished physiological responsiveness to stress, potentially associated with chronic stress exposure or dysregulation of the immune–endocrine axis (de Bruijn and Romero, 2018; Romero and Beattie, 2022). In this context, additional environmental (e.g. microhabitat temperature, resource availability, predation pressure), social (e.g. population density, social interactions, competing for reproduction) or individual-level variables (e.g. reproductive stage, parasite load, and prior exposure to stressors), rather than habitat disturbance per se, may be the primary determinants of the physiological pattern observed. Future studies incorporating parasite load, reproductive status, and microhabitat quality at different times of the activity season, and their relationship with corticosterone and leukocyte profiles, would help clarify the mechanisms underlying this unexpected result.

Like anthropogenic stressors, natural ecological stressors may also affect leukocyte profiles and baseline physiological stress. For example, energetically demanding life-history stages such as vitellogenesis, egg production, and parental care are known to modulate immune parameters, including leukocyte distributions and H/L ratios (reviewed in Davis and Maney, 2018). Reproduction, in particular, represents a major energetic investment in reptiles and often entails trade-offs between immune function and other physiological processes (French et al., 2007; Ruiz et al., 2010). Two non-mutually exclusive factors may explain the higher H/L ratio observed in the undisturbed site, which indicates higher levels of stress responses compared to the disturbed site. First, increased competition for thermal and trophic resources may arise from differences in population density, possibly higher in the undisturbed population (Brizio et al., 2025). In ectotherms, access to optimal basking sites directly influences performance, digestion, locomotion, and reproductive output (reviewed in Angilletta, 2009). Thus, intensified intraspecific competition for high-quality thermal microhabitats and food resources could elevate baseline glucocorticoid secretion and alter leukocyte profiles through chronic social and ecological stress (Moore and Jessop, 2003; Davis et al., 2008). Second, inter-site differences in reproductive frequency may also play a relevant role. In the undisturbed population, females produce two clutches per year, whereas only a single clutch has been recorded at the disturbed site (Brizio, Cabezas-Cartes, Avila, Boretto, unpublished data). This difference likely increases the energetic demands on females in the undisturbed habitat and may also intensify male–male competition for mates and territories due to more frequent reproductive events. Elevated energetic investment in females, together with higher stress levels associated with intrasexual competition in males, could therefore be underlying the unexpected pattern observed in H/L ratios.

The immune response and its specific components are highly context dependent and shaped by ecological conditions, resource availability, thermal environment, and life-history strategies (Gangloff et al., 2017; Zimmerman, 2020; reviewed in Field et al., 2022; Lamar et al., 2024). Nevertheless, consistent with most studies on Squamata (reviewed in Field et al., 2022), sex did not significantly influence innate immune function in *L. cuyumhue*. The effect of site on H/L ratio was similar in males and females, suggesting that environmental context overrides potential sex-specific physiological differences in this species. In this sense, microhabitat structure and thermal landscape differed markedly between sites inhabited by *L. cuyumhue* (Brizio et al., 2025). The undisturbed site exhibited higher vegetation height and lower operative temperatures, whereas the disturbed site was characterized by reduced vegetation height and higher operative temperatures, particularly during summer (Brizio et al., 2025). Individuals from the disturbed site exhibited higher body temperatures and selected microhabitats with reduced bare ground cover, suggesting behavioral compensation under potentially stressful thermal conditions (Brizio et al., 2025). Nevertheless, despite the fundamental role of thermal biology in reptilian physiology, the relationship between the H/L ratio and body temperature varied among sites. A significant and negative association was detected, but only at the disturbed site. In contrast, no relationship was found at the undisturbed site, indicating that the factors regulating leukocyte profiles may vary depending on environmental context. Additional research is needed to determine the mechanisms underlying this site-specific pattern.

Under the hypothesis that lizards from the disturbed site experience chronic stress, we evaluated circulating CORTs in adult males of *L. cuyumhue*. Although no significant differences in baseline CORTs were detected between sites, a significant negative correlation between CORT and the H/L ratio was observed at the disturbed site (Fig. 3). Although the number of serum samples analyzed from the disturbed site was smaller than that from the undisturbed site, this result suggests that endocrine and hematological stress parameters may not respond in parallel under these disturbed conditions. A similar response has been reported in urban populations of *U. ornatus*, in which individuals exhibited elevated leukocyte counts and lower corticosterone concentrations than rural lizards (French et al., 2008). Responses to anthropogenic disturbance in squamates appear to be context dependent (French et al., 2010). While some species exhibit elevated glucocorticoids and suppressed immune function in response to urbanization or habitat alteration, others may benefit from increased food availability, novel microhabitats, or reduced predation pressure in modified environments (French et al., 2018). Importantly, several studies in reptiles demonstrate that H/L ratios and corticosterone concentrations do not consistently covary and therefore cannot be used interchangeably as proxies of stress (Seddon and Klukowski, 2012; Romero and Beattie, 2022; Duran et al., 2024). Glucocorticoids often reflect acute or short-term endocrine responses, whereas leukocyte profiles may integrate cumulative physiological challenges over longer temporal scales (Davis et al., 2008; Davis and Maney, 2018).

Seasonality represents another major external driver of immune and endocrine variation in reptiles (reviewed in Field et al., 2022). In the present study, season significantly influenced the H/L ratio, CORT, and BCI in adult lizards. Higher H/L ratios and BCI values were recorded in spring compared to summer, and these seasonal differences were consistent across sites. Moreover, BCI was positively associated with the H/L ratio in the undisturbed site, suggesting that individuals with better energetic condition may sustain higher leukocyte investment during periods of reproductive activity. In contrast, CORT in adult males was higher in summer than in spring, a pattern also consistent across sites. Notably, the H/L ratio was affected by both season and site, whereas CORT varied only seasonally. This divergence indicates that leukocyte profiles may be more sensitive to local environmental conditions than circulating glucocorticoids. Because changes in leukocyte distributions often reflect sustained or cumulative physiological demands, spatial variation in H/L ratios may represent longer-term adjustments to site-specific ecological pressures (Davis and Maney, 2018). Conversely, the consistent seasonal increase in CORT across sites supports the interpretation that broad-scale seasonal factors, such as temperature, reproductive stage, or resource dynamics, primarily drive endocrine responses in adult males of this species. Together, these patterns highlight the value of combining BCI with hematological and endocrine parameters to characterize stress responses under natural conditions.

Consistent with the differing responses among stress indicators, the lack of a relationship between BCI and CORT at either the undisturbed or disturbed site suggests that environmental disturbance did not translate into detectable differences in energetic condition in adult males of *L. cuyumhue*. Although circulating corticosterone reflects endocrine responses to environmental challenges, BCI represents a more integrative measure of long-term energy balance, incorporating cumulative effects of food intake, metabolic expenditure, and allocation to growth and reproduction (Bonnet et al., 1998; Schulte-Hostedde et al., 2005). Glucocorticoid levels may increase in response to acute or seasonal stressors without necessarily producing measurable changes in body reserves, particularly when individuals can compensate behaviorally or when resource availability remains sufficient (Romero, 2004; Bonier et al., 2009). In disturbed habitats, reptiles may adjust activity patterns, microhabitat selection, or foraging strategies to buffer energetic costs (French et al., 2018). Such compensatory mechanisms could allow individuals to maintain a stable body condition despite endocrine activation. Consequently, variation in corticosterone associated with site conditions may not be directly mirrored by changes in BCI, highlighting a decoupling between short-term endocrine responses and longer-term measures of condition in wild reptiles (Meylan et al., 2010).

Assessing the effects of habitat alteration on the physiology, behavior, and population dynamics of threatened species is essential for designing effective conservation strategies. To our knowledge, no previous studies have addressed these questions under natural field conditions in anthropogenically disturbed habitats within the Liolaemidae family. Our findings indicate that anthropogenic disturbance is associated with changes in immune-related parameters, as reflected by differences in leukocyte profiles and H/L ratios, whereas corticosterone concentrations and body condition did not differ between disturbed and undisturbed sites and were primarily influenced by seasonal variation. Then, the immune parameters may be more sensitive indicators of anthropogenic disturbance than corticosterone concentrations or body condition in *L. cuyumhue*. The observed physiological responses to anthropogenic disturbance were parameter specific, with effects detected in immune-related variables but not in corticosterone concentrations or body condition. Further research in this species and similar systems is necessary, particularly to disentangle the mechanisms underlying the apparent decoupling between stress and immune indicators. We recognize that a limitation of our study is that CORT was measured only in adult males and that the number of serum samples available for this analysis was smaller for the disturbed site than for the undisturbed site. In addition, our results are based on single measurements obtained during the activity season, and therefore, it is not possible to analyze the temporal variation within individuals. Integrating additional physiological, ecological, and behavioral variables will also be essential to better understand how increasing hydrocarbon exploration and exploitation in Vaca Muerta (Neuquén, Argentina) may be shaping the physiology and persistence of this critically endangered species. Overall, our study represents an important first step toward understanding how large-scale environmental disturbances influence physiological regulation in natural populations and highlights the need for a cautious interpretation of commonly used stress proxies in complex and changing environments.

## MATERIALS AND METHODS

### Field work

The present study was carried out in an oil field located in the Bajo de Añelo basin (37.4° to 38.5° S and 68.4° to 69.8° W; Neuquén Province, Argentina). The study site is included within the Vaca Muerta basin, a highly productive oil and gas geological formation. The region has a temperate arid to semiarid climate, with a mean annual temperature of 14.2°C and an annual precipitation averaging 137.2 mm, concentrated primarily during winter and spring (Busso and Bonvissuto, 2009). The landscape is representative of the Monte Desert, where vegetation is dominated by perennial shrub species, particularly *Larrea* spp. (jarillas), while herbaceous plants and grasses are scarce and trees are largely absent, resulting in a structurally homogeneous shrub-steppe community (León et al., 1998; Roig et al., 2009).

For comparative purposes, we used the same two sites described in Brizio et al. (2025). Both sites share similar abiotic and biotic characteristics and are inhabited by the endemic species *L. cuyumhue* but differ in their level of anthropogenic disturbance: a disturbed site that comprises an area of 81,765 m^2^ traversed by an unpaved road and includes an oil well established in 1991 and an undisturbed site with an area of 103,194 m^2^ situated approximately 1 km from the nearest road and 8 km away from the disturbed site.

Five field trips were conducted between September 2022 and March 2023. For the analyses, the five sampling events were grouped into two austral seasons: spring and summer. A total of 53 adult individuals (*N*_♂_=34; *N*_♀_=19) were captured at the undisturbed site (*N*_♂spring_=17; *N*_♂summer=_17; *N*_♀spring_=7; *N*_♀summer_=12), and 46 adult individuals (*N*_♂_=23; *N*_♀_=23) at the disturbed site (*N*_♂spring_=13; *N*_♂summer=_10; *N*_♀spring_=5; *N*_♀summer_=18). Each lizard was captured by a sliding noose during its active period between 8:30 a.m. and 8:30 p.m. Body temperature (*T*_b_) was recorded just after capture with a digital thermometer (TES 1303; Electrical Electronic Corp., Taipei, Taiwan, China; ±0.03°C digital thermometer) connected to a thermocouple (TES TP-K01, 1.62 mm diameter), which was introduced inside the lizard's cloaca. To avoid heat transfer from the operator's hands to the lizard's body, *T*_b_ was measured within 10 s of capture. Then, within 5-min post-capture, we took a blood sample from the caudal vein at the base of the tail (e.g. Amey and Whittier, 2000; Troiano, 2013; Eymann et al., 2010) with an insulin syringe (1 cc, c/a 29G×1/2″, Terumo). To analyze the leukocyte profile, a subsample of the blood extracted was used to prepare a blood smear, which was dried by air and fixed by heat. Frozen gel packs were utilized to keep the remaining blood sample in a thermal bag for a maximum of 8 h. After that, the samples were centrifuged, and the serum obtained was stored at −20°C until corticosterone quantification. Due to the limited blood volume that could be safely collected from each individual, CORTs were determined only in adult males. In females, the available serum was allocated to the determination of estradiol and progesterone concentrations as part of a parallel study of reproductive endocrinology.

We recorded the body mass (digital pocket scale 500 g, 0.01 g), SVL (digital caliper Lee Tools, ±0.02 mm), and sex (based on the presence of pre-cloacal pores in males; Avila et al., 2009) of each lizard. The lizards were then independently placed in cloth bags until release at the end of the day (to avoid recapture). Capture sites were georeferenced with a GPS (Garmin GPS MAP 64s), and all the lizards were returned healthy to their precise capture sites.

The field work carried out for the present study was authorized by the ‘Dirección Provincial de Áreas Naturales Protegidas y Recursos Faunísticos de la Secretaría de Desarrollo Territorial y Ambiente’ of Neuquén Province (Resolution number 009/22). Besides, the Institutional Committee for the Care and Use of Laboratory or Experimental Animals (CICUAL) of INIBIOMA (Instituto de Investigaciones en Biodiversidad y Medioambiente) of CONICET, Argentina, approved the experimental procedures used for this research (protocol number 2020-030). The present work was developed in accordance with relevant guidelines and regulations, including the Guide for the Care and Use of Laboratory Animals (eighth edition; National Academies Press, Washington, DC, USA) and the rules detailed in Argentine National Law No. 14346. We did not euthanize any individual. We also considered the ARRIVE guidelines when conducting this research.

### Laboratory work

#### Analysis of blood samples

May-Grünwald Giemsa (Biopack^®^) was used to stain the blood smears because it emphasizes granulations and increases erythrocyte staining (Martínez-Silvestre et al., 2011). The leukocyte profile was expressed as a percentage of each white blood cell type, with a total count of 100 leukocytes per smear (following the methodology described in Duran et al., 2019, 2024). We considered the leukocyte categories established for reptiles by Stacy et al. (2011) and previously observed in other Liolaemidae species (Duran et al., 2019, 2024; Juárez Heredia et al., 2024) to classify the leukocytes as lymphocytes, heterophils, basophils, eosinophils, monocytes, and azurophils (Fig. 1). Also, we estimated the H/L ratio. A single operator (F.D.) analyzed all the slides under an optical microscope (Olympus^®^ BX51, America Inc., Melville, NY, USA; 1000× with immersion oil) equipped with a camera (Tucsen^®^ DigiRetina 16; 16 MP CMOS sensor).

The CORTs in males (*N*=40) were determined by an enzyme immunoassay (EIA; reagents were obtained from the University of Veterinary Medicine, Veterinärplatz 1, A-1210 Vienna, Austria; Palme and Möstl, 1997) following the methods described in Duran et al. (2024): (a) initially, every serum sample was mixed with ethyl ether at a 1:10 v/v ratio, afterwards agitated for 10 s and centrifuged for 15 min at 2600 ***g***; (b) a thermostatic bath at 40°C was used to evaporate the supernatant that contained the solvent; (c) the solvent was then reconstituted in EIA buffer solution (1:10 dilution); (d) each sample was analyzed in duplicate in an Ivdiagnostik M201 microplate reader, presenting a coefficient of variation lower than 10%. The hormonal determinations were carried out in the laboratory of Dr. Juan Manuel Busso at the Instituto de Investigaciones Biológicas y Tecnológicas (National University of Córdoba-CONICET, Córdoba, Argentina).

#### BCI

The scaled mass index (*M)* was used to estimate the *BCI* of each individual. This index represents a good estimator of the stored (fat) energy and was developed by Peig and Green (2009, 2010). It is calculated by the following formula:

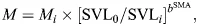
where *M_i_* and SVL*_i_* are each individual's body mass and SVL, SVL_0_ is the population's SVL arithmetic mean, and the *b*^SMA^ exponent represents the standardized major axis slope from the regression of ln mass on ln SVL for the population (Peig and Green, 2009, 2010) and was calculated using the package ‘lmodel2’ (Legendre, 2015) in R (R Core Team, 2023).

### Statistical analyses

The statistical software SigmaPlot 14.0^®^ and R (R Core Team, 2023) were used for the data analysis of this study. Shapiro–Wilk and Levene tests were applied to confirm that the assumptions of normality and homoskedasticity were met. In the cases where these assumptions were not satisfied, the corresponding nonparametric tests were carried out. Differences in leukocyte percentage between disturbed and undisturbed sites were assessed using appropriate comparative tests (SigmaPlot 14.0^®^).

The H/L ratio was analyzed using a GLM with a Gamma distribution and log link function, including site, season, and sex as predictors (R Core Team, 2023). Also, Pearson correlations or simple linear regressions were used to evaluate associations between H/L ratio and body temperature (*T*_b_) at each site (SigmaPlot 14.0^®^).

To evaluate associations between CORT and morphometric or thermal variables (SVL, body mass, and *T*_b_), simple linear regressions or Pearson correlations, as appropriate, were performed (SigmaPlot 14.0^®^). Spearman rank correlations were used to assess the association between CORT and individual leukocyte types at each site when the assumptions of normality were not met. The CORT was analyzed using a GLM with a Gamma distribution and a log link function, including site, season, and H/L ratio as predictors (R Core Team, 2023). The best-fit model included season and the interaction between site and H/L ratio.

The BCI was analyzed using a GLM with a Gamma distribution and log link function, including site and season as predictors (R Core Team, 2023).

For each of the three GLMs, model selection was performed using a stepwise selection procedure. We began with models that included the explanatory variables and sequentially evaluated the contribution of each predictor using LRTs comparing nested models. Variables that did not significantly improve model fit (*P*>0.05) were removed. We also evaluated two- and three-way interactions among predictors. When interaction terms were not significant and did not improve model fit, they were excluded from the final model. Model selection was based on LRTs and supported by comparisons of Akaike's information criterion, favoring the most parsimonious model.

Associations between physiological variables (e.g. BCI versus H/L ratio and BCI versus CORT) were examined using simple linear regressions or Pearson correlations, as appropriate (SigmaPlot 14.0^®^), for the pooled dataset and separately by site. Data are presented as the mean±s.e. or median, and statistical significance was set at *P*<0.05.
